# A Customized Microfluidic Paper-Based Platform for Colorimetric Immunosensing: Demonstrated via hCG Assay for Pregnancy Test

**DOI:** 10.3390/bios11120474

**Published:** 2021-11-25

**Authors:** Mohammad Rahbar, Siyi Zou, Mahroo Baharfar, Guozhen Liu

**Affiliations:** 1 Graduate School of Biomedical Engineering, The University of New South Wales, Sydney, NSW 2052, Australia; rahbar2007@yahoo.com (M.R.); m.baharfar@unsw.edu.au (M.B.); 2 School of Life and Health Sciences, The Chinese University of Hong Kong, Shenzhen 518172, China; siyizou@link.cuhk.edu.cn

**Keywords:** microfluidic paper-based analytical devices, point-of-care testing, human chorionic gonadotrophin, colorimetric detection, immunosensing, pregnancy test

## Abstract

Over the past decades, paper-based lateral flow immunoassays (LFIAs) have been extensively developed for rapid, facile, and low-cost detection of a wide array of target analytes in a point-of-care manner. Conventional home pregnancy tests are the most significant example of LFAs, which detect elevated concentrations of human chorionic gonadotrophin (hCG) in body fluids to identify early pregnancy. In this work, we have upgraded these platforms to a higher version by developing a customized microfluidic paper-based analytical device (μPAD), as the new generation of paper-based point-of-care platforms, for colorimetric immunosensing. This will offer a cost-efficient and environmentally friendly alternative platform for paper-based immunosensing, eliminating the need for nitrocellulose (NC) membrane as the substrate material. The performance of the developed platform is demonstrated by detection of hCG (as a model case) in urine samples and subsequently indicating positive or negative pregnancy. A dual-functional silane-based composite was used to treat filter paper in order to enhance the colorimetric signal intensity in the detection zones of μPADs. In addition, microfluidic pathways were designed in a manner to provide the desired regulated fluid flow, generating sufficient incubation time (delays) at the designated detection zones, and consequently enhancing the obtained signal intensity. The presented approaches allow to overcome the existing limitations of μPADs in immunosensing and will broaden their applicability to a wider range of assays. Although, the application of the developed hCG μPAD assay is mainly in qualitative (i.e., positive or negative) detection of pregnancy, the semi-quantitative measurement of hCG was also investigated, indicating the viability of this assay for sensitive detection of the target hCG analyte within the related physiological range (i.e., 10–500 ng/mL) with a LOD value down to 10 ng/mL.

## 1. Introduction

Traditional lateral flow (immuno) assays (LFIAs) are immunochromatographic paper-based platforms comprising a nitrocellulose (NC) membrane as a reaction surface with a high protein-binding capability, suitable for irreversible antibody immobilization. The NC membrane is impregnated with the corresponding capture antibodies at the specific zones, indicating the test-line and control-line for the qualitative or semi-quantitative detection of specific target analytes [1,2]. For example, conventional home pregnancy test LFAs are the most popular and widely commercialized tools for rapid, simple, and qualitative detection of elevated concentrations of human chorionic gonadotrophin (hCG) in urine or serum samples, identifying pregnancy at early stages. hCG is a glycoprotein hormone normally generated by the placenta during pregnancy. hCG molecule is composed of 237 amino acids with a molecular mass of 36.7 kDa and two subunits, the alpha and beta. Secretion of hCG starts almost a week after fertilization, which reaches to its peak (1000 ng/mL) in 8 weeks of pregnancy. After this period, the hCG level begins to decline until it stabilizes. The normal serum hCG level is less than 10 ng/mL; the detection of hCG at this level is thus significant for early pregnancy identification. Although there are various methods available for detection of hCG (e.g., ELISA), the conventional LFA based tests offer numerous advantages, such as being rapid, simple, low cost, and easy to operate [3].

Due to the natural strong affinity for proteins, the NC membrane has been traditionally used as the most favorable reaction surface for LFAs. However, there are some issues and limitations associated with the NC membrane such as the high cost, fragility, flammability, toxicity (alteration to habitats), and hydrophobicity (requires deposition of surfactants). In the past decades, there have been extensive efforts made in the LFA industries in order to replace the NC membrane with other possible alternative materials, including nylon, polyvinylidene fluoride (PVDF), polyethersulfone, polyethylene, fused silica, and other composite membranes. However, each of these materials has exhibited some challenges and drawbacks, thereby maintaining the superiority of the conventional NC membrane [4,5]. These materials are relatively costly, incompatible with the chemistry of performed bioassays, and also present a lower signal-to-noise ratio compared to NC membrane. Nevertheless, a fluorescent-based LFA was recently reported for detection of cardiac troponin I, where the filter paper was modified with carbon nanofibers and used instead of NC membrane [6].

In 2007, the Whitesides group expanded the concept of paper-based analytical devices and introduced microfluidic paper-based analytical devices (μPADs) as a new generation of paper-based point-of-care (POC) platforms [7]. They demonstrated that filter paper can be used as a viable platform for development of disposable analytical devices, presenting numerous advantages over the conventional materials utilized in the fabrication of such devices, particularly NC membranes [8]. Filter paper is a low-cost, accessible, hydrophilic, flexible, and disposable material, which makes it an ideal platform for POC (bio)chemical diagnostic testing. The emerging μPADs use patterned filter paper as a platform for POC testing, providing a great deal of flexibility for qualitative, semi-quantitative, or quantitative measurements of a wide variety of target analytes in diverse sample matrices [9,10]. We also developed a μPAD for detection of human serum albumin based on smart-phone signal readout [11]. Despite of all these benefits as well as the significant progress made in the realm of μPADs, the lack of enough protein binding capacity as one of the main limitations of the filter paper has narrowed down the application of μPADs to only some simple (bio)chemical assays where there is no need for immobilized antibodies or proteins typically required in traditional sandwich LFAs (e.g., hCG pregnancy test) [12]. Essentially, this is due to the inert nature, hydrophilicity, and neutral surface chemistry of filter paper fibers, not providing the required attraction forces to retain the antibodies, whereas the existing hydrophobic and electrostatic interaction forces allow retention of biomolecules in NC membranes. There have been some strategies reported to address this limitation, presenting different types of chemical or physical paper modification techniques to retain various (bio)chemical reagents upon paper [13,14,15,16,17,18,19,20] and also various signal amplification strategies [21,22] on paper. However, the presented methods are not usually practical enough to selectively immobilize the capture antibodies in the designated detection zones upon the surface of filter paper, enabling development of sequential multistep sandwich immunoassays. Majority of the introduced paper treatment methods are not efficient enough for selective modification of the desired detection spots upon paper by many tokens, such as involving the entire paper surface area in modification, which is not favorrable and can often cause non-specific binding and affecting the overall assay performance. In addition, these methods usually involve some tedious modification steps requiring multiple times exposure of paper to the treatment solutions, which consequently affects the 3D network of paper and deteriorates the natural capillary-based fluid flow along the paper channels [13,14,15,16,17,18,19]. For instance, Zhu et al. modified paper via oxidation of cellulose fibers using NaIO_4_ solution; the entire μPAD was immersed and left in this solution for about 40 min to generate aldehyde groups on the surface of cellulose. Afterwards, μPAD was washed thoroughly with water and then dried for future use [18]. In another work, various chemistries were investigated for chemical modification of filter paper for immobilization of biomolecules. Among all, modification with KIO_4_ was found to be the most efficient method; however, it involved incubation of the whole filter paper in aqueous solution of KIO_4_ at 65 °C for 2 h [23]. After, the modified paper was washed three times with water and then blotted and dried overnight. Finally, the modified paper was used for fabrication of μPADs. 

In the present work, a customized μPAD was proposed as an alternative to the conventional LFA-based colorimetric immunosensing systems. The performance of the developed μPAD assay was demonstrated via qualitative and semi-quantitative detection of hCG protein in urine samples, indicating positive or negative pregnancy. This was achieved through selective immobilization of the desired capture antibodies upon the detection zones, where filter paper was impregnated with a dual-functional carboxylated silane composite, allowing formation of the sandwiched colorimetric complexes. The design, dimension, and geometry of the platform has been also optimized generating a regulated fluid flow to enhance the intensity of the obtained colorimetric signal. Both presented optimizations in surface chemistry of filter paper and also the device design, enabled detection of the target hCG protein in the tested samples and subsequently corelating them with either a positive or negative pregnancy. In addition, the presented optimized filter paper modification methods along with the new functional design of the device are superior to the previously reported works, which can be used in the future for similar applications and analytes. This will broaden the application of μPADs to a wider range of assays while eliminating the need for the NC membrane as the dominant material used for paper-based immunoassays.

## 2. Experimental Section

### 2.1. Chemicals and Materials

All chemicals were of analytical reagent grade. Human chorionic gonadotropin (hCG) protein, goat anti-alpha hCG monoclonal capture antibody, and goat anti-mouse IgG antibody, were all supplied from Sapphire Bioscience Pty. Ltd. (Redfern, NSW, Australia). Mouse anti-beta hCG monoclonal antibody-colloid gold (AuNPs) conjugate was purchased from Abcam (Melbourne, Australia). Phosphate buffer saline (PBS) (0.01 M, pH 7.4), sucrose, tween-20, tris, and bovine serum albumin (BSA), were purchased from Sigma–Aldrich (Castle Hill, NSW, Australia). Water was treated with a Millipore (Bedford, MA, USA) Milli-Q water purification system and was used throughout. Whatman grade 4 qualitative filter paper with a pore size of 25 µm and thickness of 210 µm (GE Healthcare Australia Pty. Ltd., Parramatta, NSW, Australia) was used for fabrication of µPADs. Herein, the relatively large paper pore size (i.e., 25 µm) facilitates full transport of AuNPs probe through paper channels. Glass fiber membrane (SB06), absorbent pad, and backing pad were purchased from Shanghai Kinbio Tech. Silica gel particles (40–63 µm) were obtained from Silicycle (Quebec, QC, Canada). Silane-Polyethylene glycol (PEG)-carboxylic acid (COOH) was purchased from Nanocs Inc. (New York, NY, USA). The Freedom pregnancy test kit (Chemist Warehouse, Sydney, NSW, Australia) was used to test the real urine samples. Human chorionic gonadotropin (hCG) protein was purchased from Nanjing Santa Scott biotechnology, 0.01 M pH 7.4 PBS, Diagnostic kit for human chorionic gonadotropin (colloidal gold immunochromatography) was purchased from Beijing Zizhu pharmaceutical.

### 2.2. Instrumentation

Silhouette Studio^®^ (V4.1.156) free software was used to design the microfluidic layouts. A wax printer (Colorqube 8870, Xerox, Norwalk, CT, USA) was utilized to print the microfluidic patterns upon the filter paper. A regular desktop Epson scanner was used to acquire images of µPADs. The quantitative image analysis was carried out using free image j software. A revolving handheld puncher was used to cut out circles on the paper. A scanning electron microscopy (SEM, JEOL InTouchScope, JSM-IT 500 HR, Frenchs Forest, NSW, Australia) coupled to an energy-dispersive X-ray spectroscopy (EDS, JEOL, Ex-74600U4L2Q, Frenchs Forest, NSW, Australia) were used for investigation of morphology and elemental analysis of paper device.

### 2.3. Design and Fabrication of µPADs

#### 2.3.1. Wax Printing

**Figure 1:**
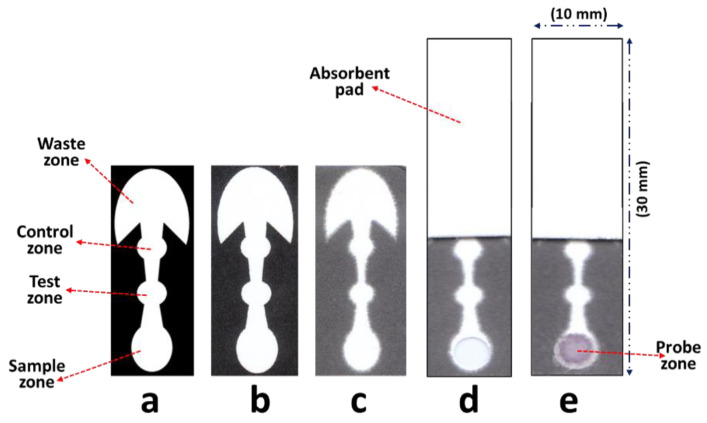
Fabrication process of µPADs. (**a**) Computer layout comprising detection, sample, and waste zones. (**b**) Printed pattern on the paper. (**c**) Melted wax. (**d**) Sample zone punched out and absorbent pad stacked on the waste zone. (**e**) Probe disk is stock on the sample zone.

The microfluidic patterns were designed using the Silhouette Studio^®^ and wax printed on the paper as reported previously [24,25]. The pattern was composed of a sample zone, test zone (T), control zone (C), and a waste zone. The outline and dimensions (before and after wax melting) of the µPADs are illustrated in Figure 1a. The printed paper was then placed on a hot plate (2 min/130 °C) to melt the wax through the thickness of the filter paper. The fabrication process was continued by pipette drop-casting of the corresponding reagents upon the desired zones. 

#### 2.3.2. Reagent Deposition

First, the T and C zones were treated with the silane composite (2 µL, suspension was shaken before pipetting) and then incubated in an oven (37 °C/30 min). After, the required antibodies were deposited into the T and C zones (anti-alpha hCG and anti-mouse IgG respectively, 1.5 µL of 1 mg/mL in each zone) and then incubated further in the oven (37 °C/1 h). Finally, the blocking buffer (PBS pH = 7.4 containing BSA 1% *w*/*v*, Tween-20 0.25% *v*/*v*, sucrose 2%) was loaded (total 5 µL in 1 µL increments) over the entire area of μPADs (excluding the waste zone) and incubated for another 1 h (37 °C). The number of exploited reagents was optimized in order to find out the least amount required while generating the highest assay sensitivity. 

#### 2.3.3. Final Assembly

After all the paper treatment steps, the μPADs final assembly was accomplished by punching out the sample zone area using a handheld puncher, creating a hole (diameter 5 mm) to affix the probe disk to the μPADs. Then, paper was attached to the sticky side of a backing pad providing further mechanical support. In the next step, a rectangular shaped layer of absorbent pad (10 × 20 mm) was stacked over the waste zone in order to generate extra wicking capacity, allowing larger volumes of samples being tested. Then, a probe disk was attached to the sample zone area. To prepare the probe disk, a circular disk (6 mm) was punched out from the glass membrane and then it was treated initially with the blocking buffer (5 µL) followed by addition of Anti-hCG-gold conjugate (5 µL) while being incubated in the oven (37 °C/30 min) for each treatment. The disk size (6 mm) was slightly larger than the hole (5 mm) in order to make sure that the entire area in the sample zone is covered by the disk. Prepared µPADs were kept in the fridge (4 °C) for future use. Photographs of µPADs throughout different preparation steps are shown in Figure 1. 

### 2.4. Preparation of Silane Composite

100 mg silica gel particles (60 µm) and 3 mg silane−PEG−COOH were dispersed in 1 mL of ethanol (50% *v*/*v*) and incubated for 24 h (60 °C). Then the suspension was centrifuged and washed three times with ethanol (50% *v*/*v*). Afterward, the precipitate was redispersed in 500 µL PBS and then 200 µL of a 1:1 solution of EDC/NHS (10 mg/mL in PBS) was added and incubated further for 2 h in room temperature. Finally, the suspension was centrifuged and washed with PBS. The precipitate was eventually resuspended in 500 µL PBS and kept in dark for further use (always used in the same day).

### 2.5. Analysis Procedure

The hCG standard solutions were prepared by diluting a stock solution (100 μg/mL) of hCG in buffer solution (PBS with BSA 0.1% *w*/*v*). The testing was performed by loading 50 μL of hCG solution at different concentration levels (0–500 ng/mL) into the sample zone of µPADs. For the qualitative measurements, formation of a visible red color pattern at the T zone indicated a positive signal, while absence of the color was associated with the negative signal. The semi-quantitative signal readout was carried out after 20 min (stabilized color intensity) by scanning the µPADs using a desktop scanner followed by image analysis using ImageJ software. Further details in this regard are provided in the following sections. 

## 3. Results and Discussion

### 3.1. Working Principle of the hCG µPAD Assay

**Figure 2:**
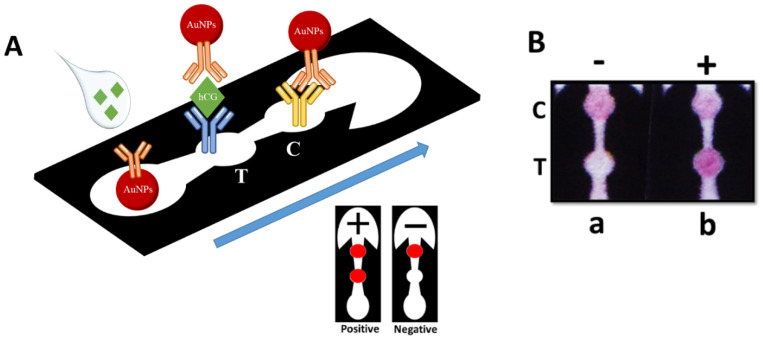
(**A**) Schematic representation of hCG µPAD assay working principle: hCG analyte will bind to the anti-beta hCG antibody conjugated on AuNPs. The hCG−probe complex will move toward the T and C zones. The formed complex will be captured at the T zone by immobilized hCG alpha antibody. Free excess probe will travel further along the microfluidic channels until they reach the C zone, where they are captured by the anti-IgG antibody. (**B**) Actual photographs of hCG µPAD assay presenting both the positive and negative responses to hCG analyte.

Similar to LFA-based pregnancy tests, the presented µPAD assay is also a sandwich immunoassay, implementing AuNPs-antibody (i.e., anti-beta hCG monoclonal antibody) conjugates as the colorimetric signaling probe. As shown in Figure 2A, after loading the sample (50 μL), including hCG target protein into the sample zone, the deposited probe will be detached from the glass membrane substate and the hCG-probe complex will be formed, which will then move toward the T zone via the capillary effect. At the T zone, the complex will be captured by the immobilized hCG alpha antibody while the free probes will move even further, where they are captured by the IgG antibody present at the C zone. The appearance of two red circular pattern indicates a positive response, whereas if no hCG analyte is present in the introduced sample (i.e., no complex formation), there will not be any colored T zone, indicating a negative response.

As shown in Figure 1d,e, the filter paper at the sample zone was intentionally removed and replaced with the glass membrane disks to provide a more efficient and inert environment for hosting AuNPs probe because the glass membrane will not retain AuNPs permanently, as opposed to the filter paper. Essentially, since filter paper can strongly retain AuNPs, the particles will get stuck to paper fibers (i.e., after depositing and full drying) and consequently cannot move toward the detection zone after the sample addition. The latter issue makes paper an ineffective platform for such sequential bioassays, requiring an optimum probe fluid flow along the assay platform. It is worthwhile to mention that although AuNP has been used as the most popular colorimetric probes (signaling tags) in the space of LFAs [26]; however, due to the above-mentioned challenge, their application in µPAD based bioassays has not been explored enough due to the inconsistent and limited AuNPs transport through the filter paper network. In the presented hCG µPAD assay, this challenge was overcome simply by using a small glass membrane disk providing a proper substate for storage of probe, meanwhile allowing complete release and transport of AuNPs after sample loading. In addition, the waste zone design was also optimized in order to provide the maximum absorption capacity along with a sufficient fluid flow rate. According to the previous reports [27,28,29], a 270° fan-shaped paper compartment (Figure 1a) was incorporated to the end of µPAD as a waste zone to maximize the flow rate and avoid the natural decay in flow rate over the assay time by a continuous increase in fluid front area. An extra layer (Figure 1e) of absorbent pad (rectangular) was also stacked over the fan-shaped waste zone to generate additional wicking capacity allowing full transport of the sample and also probes along the µPAD channels, and consequently enhancing signal intensity. 

### 3.2. Paper Treatment at the Detection Zones

As mentioned in the introduction, the NC membrane has been the most widely used substate for development of LFAs due to its natural strong protein binding affinity, which makes it an ideal material for immobilization of antibodies, proteins, and other biomolecules. This is the main weakness of filter paper (used in µPADs) compared to NC membrane as it does not provide such a significant anchoring point for retention of typical capture antibodies along the detection channel of immunoassay platforms [30,31,32]. 

**Figure 3:**
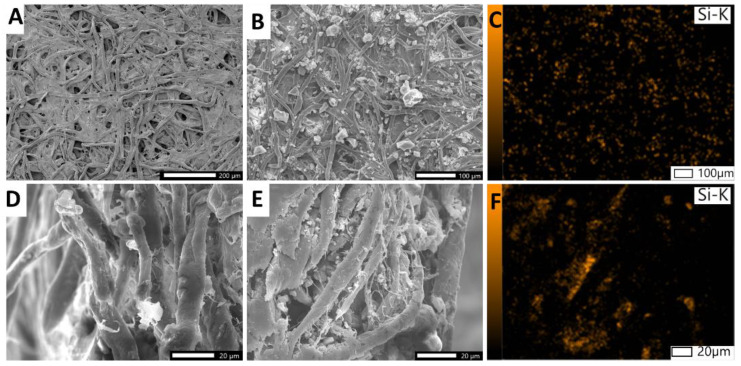
SEM images of filter paper at the detection zone of µPADs. (**A**,**D**) unmodified paper, top and cross-sectional view respectively. (**B**,**E**) treated paper, top and cross-sectional view respectively. (**C**,**F**) EDS images of treated paper, top and cross-sectional view respectively.

To overcome the above-mentioned challenges, we used a dual-functional silane-based composite to selectively treat the detection spots (i.e., only T and C zones) upon the paper substate. This composite was prepared by conjugating silica gel particles with a carboxylated compound (silane−PEG−COOH). The large silica gel part (40–63 µm) functions as a filler occupying the paper pores at the detection zones, which results in formation of a colorimetric signal with higher intensity at the surface of detection zones. We noticed that due to the large paper pore sizes (i.e., 25 µm), the immobilization of antibodies alone is not enough to generate a visible and strong colorimetric signal since most of the formed probe−hCG complex will penetrate through the thickness of paper rather than remaining on the surface of T zone. This is not a challenge in other porous substates such as NC membrane with smaller pore sizes (~1 µm) and higher density. Although, this might not be an issue while testing samples with high concentrations of hCG analyte, it is problematic in the case of dealing with low concentrations of analyte (i.e., low amount of hCG-probe complex), where a highly sensitive response is required. Therefore, the pores must be filled as much as possible (i.e., not fully block the channels) in order to increase the density at the detection zone and prevent the loss of the formed colored complex through the paper pores. The SEM and EDS images of the filter paper impregnated with the composite are shown in Figure 3, indicating physical entrapment and immobilization of the composite crystals in the 3D structure of paper fibers. The EDS images also indicate the distribution of Si throughout the paper fibers at the modified zones.

**Figure 4:**
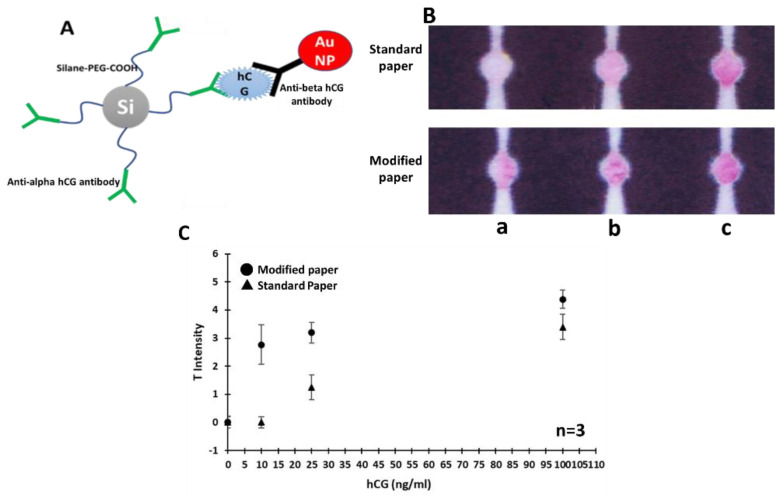
(**A**) Schematic representation of the sandwich structure of Silane composite−cap−ture antibody−(hCG−probe complex). (**B**) Photographs of µPADs made with standard and modified filter paper in detection of hCG samples with three different concentrations ((a: 10, b: 25, c: 100 ng/mL). (**C**) Semi-quantitative analysis of the obtained signal intensity from the tested µPADs. The error bars represent the standard deviations corresponding to the average values (n = 3).

On other side, the carboxylated part of the composite was responsible for retaining the capture antibodies in the detection zones, although the standard unmodified filter paper has also a weak adsorption capacity for antibodies and retains them to some extent. This is an additional step to generate more binding sites in the detection zones enhancing the antibody immobilization on the paper and ensuring a much higher sensitive signal. Basically, in treated paper after loading and incubating the antibodies into the detection zones, they will covalently bind to the activated COOH sites of the available composite and will be immobilized for further assay interactions. Therefore, this composite presents two functionalities, including a filler via the silica part and an antibody immobilizer via the COOH part. The sandwich structure consisted of the dual-functional composite, immobilized capture antibody, and hCG−probe complex is schematically depicted in Figure 4A. The effect of paper modification on signal intensity was also investigated by testing the performance of prepared µPADs with and without treatment in detection of hCG at three different concentration levels (10, 25, 100 ng/mL). Results are shown in Figure 4B,C, indicating the significant effect of modification on the obtained colorimetric signal in low concentrations of hCG. As mentioned earlier, in high concentrations the modification does not have a significant impact since the amount of available colored hCG−probe complex is large enough to generate a visible signal, while in low concentrations (i.e., 10 ng/mL, Figure 4B(a)) no signal can be acquired without paper treatment. Therefore, in order to reach the target sensitivity (i.e., 10 ng/mL) within required physiological ranges, the paper treatment is necessary.

### 3.3. Effect of Device Geometry on the Signal Intensity

**Figure 5:**
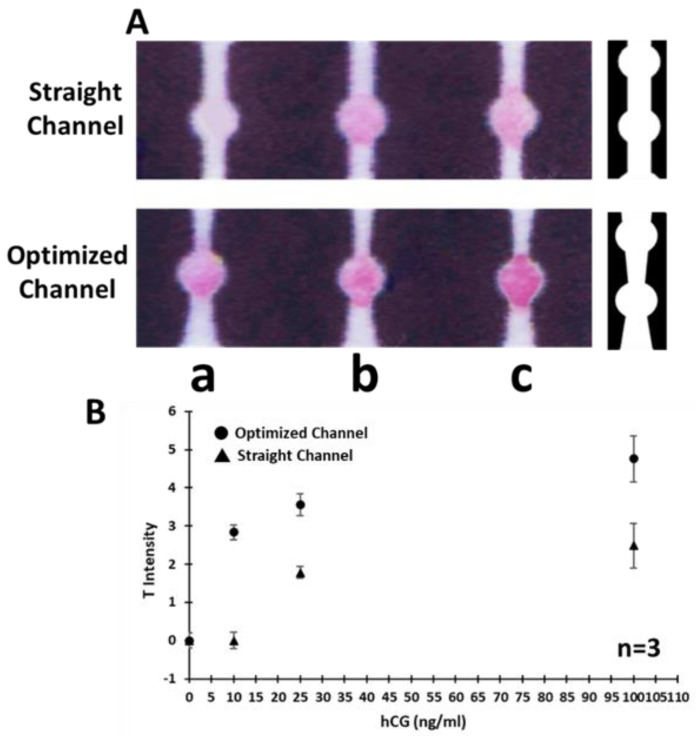
Effect of microfluidic channel geometry on the signal intensity. (**A**) Photographs of µPADs with straight and optimized channels used for hCG detection in three different concentrations (a: 10, b: 25, c: 100 ng/mL). (**B**) Semi-quantitative image analysis of tested µPADs indicating the enhancement in signal intensity after optimization of design. The error bars represent the standard deviations corresponding to the average values (n = 3).

In addition to the presented paper treatment strategy, the design of µPAD was also optimized to generate the highest signal intensity. Asymmetric (trapezoidal) [33] microfluidic connecting channels were used to regulate the sample flow rate with a desirable pattern. A trapezoidal channel with a descending width was designed to connect sample zone to the T zone while a channel with an ascending width was incorporated to connect T zone to the C zone (Figure 1c). The flow rate is relatively slower at the end and beginning of the descending and ascending channels, respectively. This combination of geometries will function as a valve, which causes a delay in fluid flow exactly at the T zone. The created delay provides more time for the hCG−probe complex to interact with the capture antibody immobilized at the T zone and consequently enhances the colorimetric signal intensity. However, this artificial delay does not significantly impact the overall assay time (20 min) as it was close to that in the µPADs with straight channels (i.e., 15 min). As mentioned earlier (Section 3.1), implementation of the fan-shaped waste zone along with an extra layer of absorbent pad at the end of µPADs (Figure 1e), maximizes the flow rate over the assay time while the delay occurs only at the desired T zone. The effect of channel geometry on the performance of the µPADs was also investigated by comparing straight channels with optimized channels. Results are shown in Figure 5, indicating the significant enhancement of signal intensity after using trapezoidal channels. 

### 3.4. Semi-Quantitative Measurement of hCG via µPADs

**Figure 6:**
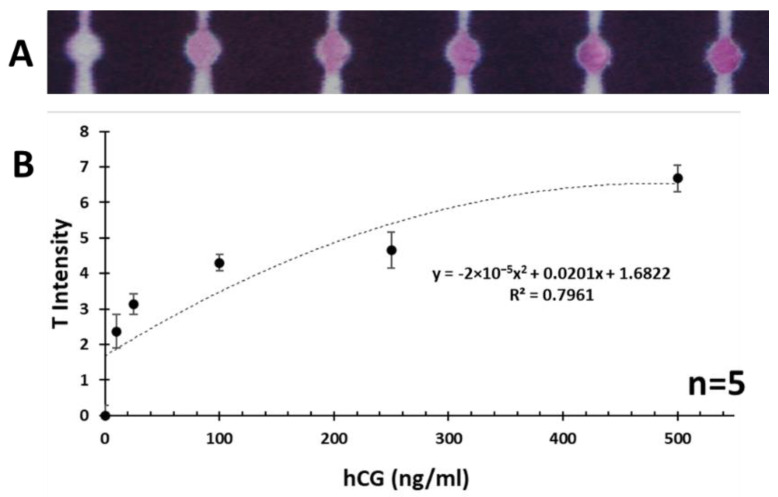
(**A**) Photographs of µPADs indicating the obtained colorimetric signals at the T zone after introducing 50 µL of standard hCG solutions in the range (0, 10, 25, 100, 250, 500 ng/mL). (**B**) Corresponding response calibration curve was acquired from quantitative image analysis of tested µPADs. A polynomial fitting model was used. The error bars represent the standard deviations corresponding to the average values.

Even though the main objective of the present work was to develop a qualitative (yes or no response) µPAD assay for pregnancy testing, the semi-quantitative measurement of hCG was also carried out to find out the dynamic range of the developed assay. This will provide a further insight on the performance of the assay and also will enhance its applicability for real world tests. The hCG standard solutions were prepared by diluting a stock solution (100 μg/mL) of hCG in buffer solution (PBS with BSA 0.1% *w*/*v*). The semi-quantitative measurement was performed by loading 50 μL of hCG solution at different concentration levels (0–500 ng/mL) into the sample zone of µPADs. It was observed that below the concentration of 10 ng/mL, there was no visible red color pattern formed at the T zone so this was considered as the detection limit, which could easily be distinguished from the background via naked eye visualization. The semi-quantitative signal readout was carried out after 20 min (stabilized color intensity) by scanning the µPADs using a desktop scanner followed by image analysis using ImageJ software. The normalized gray intensity was calculated by converting the color image into a grayscale one and then splitting it into individual RGB colors and then considering and summing the green (G) and blue (B) intensities of each data point to generate the signal or T zone intensity. A calibration chart was obtained by plotting the sum of mean normalized gray intensity of G and B components versus concentration of the hCG analyte. The calibration curve along with the photographs of µPADs are provided in Figure 6. Similar to the blank sample (0 ng/mL), samples with hCG concentration less than 10 ng/mL (e.g., 5 or 1 ng/mL) presented no visible signal so they were excluded from this curve to avoid confusion. Based on the obtained results, the proposed assay can effectively be utilized for semi-quantitative measurement of hCG within the related physiological range (10–500 ng/mL) with relative standard deviation values lower than 8.5. Meanwhile, the obtained sensitivity (10 ng/mL) is comparable with the previously reported POC colorimetric assays for detection of hCG [3]. To further evaluate the feasibility of developed assay, the commercial pregnancy test strips were applied to test the same concentration range of hCG. As shown in a set of representative photographs in Supplementary Information (SI) Figures S1–S4, the relative test line (T line) responded to the corresponding hCG concentration. The gray value of commercial test strip red band on T line was semi-quantitatively related to the concentration of hCG in the range of 10–500 ng/mL (SI Figure S3). After the double logarithmic coordinate treatment, there was a good linear relationship between the intensity and hCG concentration (SI Figure S4). The detection results were the same as that for our developed µPAD assay, indicating the cellulose filter paper-based immunoassay demonstrated the great potential for hCG detection in the future. The cost for a commercial hCG test strip is about RMB 2, but one µPAD strip costs about RMB 0.5 considering the cost of a cellulase paper is RMB 0.5 (1 piece of filter paper with a diameter of 15 cm) can make about 40 test strips) and the cost of reagents on a test strip. Thus, our developed hCG test strip is more cost-effective and environmentally friendly POCT for pregnancy testing comparing that to traditional pregnancy tests.

### 3.5. Real Sample Analyses

**Figure 7:**
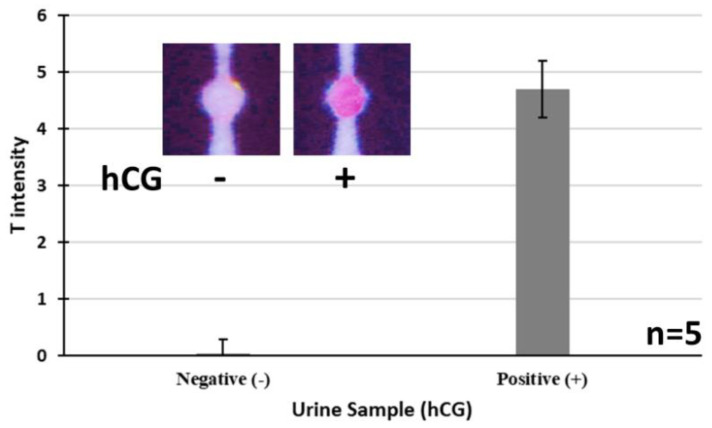
Response of the developed assay to real urine samples acquired from semi-quantitative image analysis. The error bars represent the standard deviations corresponding to the average values. Photographs of the used µPADs for real sample tests are also shown in the inset, indicating the negative and positive signals.

After establishing the hCG µPAD assay in PBS, the performance of assay was tested in real clinical samples. Urine samples were acquired from two volunteer female cases who were known to be positive and negative for pregnancy. Informed consent was obtained under approved Human Research Ethics Committee protocols (HREC Committee B, HC190300 approval number). The samples were tested (50 µL, directly with no treatment or preparation) for pregnancy using the µPADs, which indicated the expected results for each sample (negative vs. positive). As expected, the intensity of test zone for the positive sample was significantly stronger than that in the negative sample, and corresponds to about 120 ng/mL hCG according to the calibration curve in Figure 6. In order to validate the obtained results, the commercial Freedom pregnancy test kit was used in parallel to test the urine samples, which illustrated a perfect agreement with the developed µPAD assay. These results indicate the applicability of the developed µPAD assay for detection of pregnancy in real clinical samples. These results are shown in Figure 7, illustrating the response of the developed assay to real urine samples acquired after qualitative image analysis of the used µPADs.

## 4. Conclusions

In this work, a specially designed µPAD was successfully developed for colorimetric point-of-care qualitative and semi-quantitative immunosensing, demonstrated by determination of hCG in urine samples. The performed qualitative and semi-quantitative hCG analysis and obtained results indicated the viability and applicability of the developed platform for further POC testing applications. The proposed methodology can make a positive contribution to the point-of-care testing market by expanding the applicability of μPADs and their reliability for clinical diagnostics in real world scenarios, providing a more cost-effective and environmentally friendly platform for immunosensing applications. The presented functional and useful features of the developed platform, including effective paper treatment strategy along with a functional µPAD design, can be investigated further for potential applications in point-of-care detection of significant biomarkers in low-resource settings. In order to improve the sensitivity limitation associated with POC testings, applying CRISPR/Cas biosensing on a µPAD can be explored [34,35,36]. Furthermore, the colorimetric feature of this assay allows further developments for targeting smartphone-based semi-quantitative POC detections well-suited for on-site measurements using widely accessible gadgets and tools [11].

## Data Availability

Not applicable.

## Supplementary Materials

The following are available online at https://www.mdpi.com/article/10.3390/bios11120474/s1, Figure S1. Images of commercial test strips for detection of hCG in different concentrations (n = 3). Figure S2. Images of commercial test strips for detection of hCG in the concentration of 0 ng/mL and 500 ng/mL, respectively. Figure S3. A calibration curve drawn of T line gray values in the range of 0 to 500 ng/mL. Figure S4. A calibration curve X&Y axis were calculated by double logarithmic coordinates.
