# Supplementary material for: A Customized Microfluidic Paper-Based Platform for Colorimetric Immunosensing: Demonstrated via hCG Assay for Pregnancy Test
Source: Biosensors (Basel). 2021 Nov 25;11(12):474. doi: 10.3390/bios11120474 (PMC8699738; doi:10.3390/bios11120474)
Supplement: Supplementary file 1 [file biosensors-11-00474-s001.zip › biosensors-1432664-supplementary.pdf]

biosensors

MDPI

Supplementary Materials

A Customized Microfluidic Paper-Based Platform for Colorimetric Immunosensing: Demonstrated *via* hCG Assay for Pregnancy Test

Mohammad Rahbar [1], Siyi Zou [2], Mahroo Baharfar [1] and Guozhen Liu[1,2,*]

[1] Graduate School of Biomedical Engineering, The University of New South Wales, Sydney NSW, 2052, Australia
[2] School of Life and Health Sciences, The Chinese University of Hong Kong, Shenzhen, 518172, China
* Correspondence: liuguozhen@cuhk.edu.cn

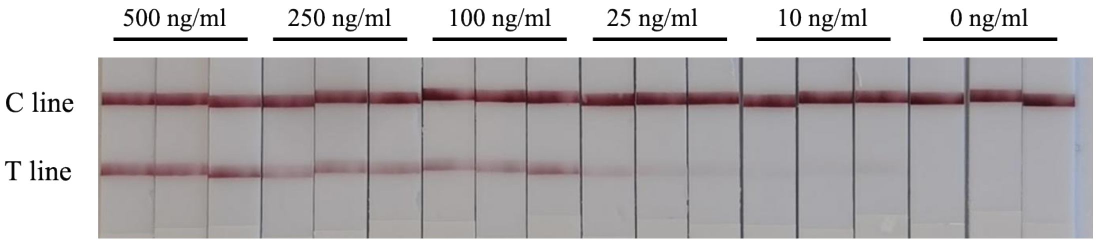

Figure S1. Images of commercial test strips for detection of hCG in different concentrations (n = 3).

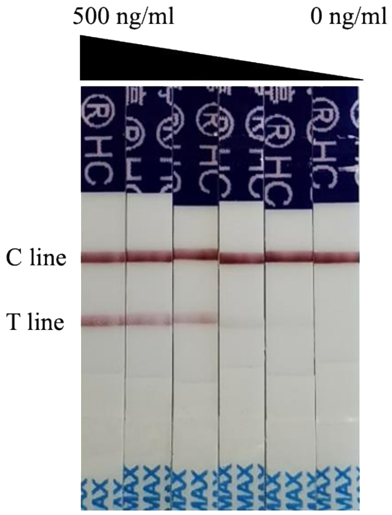

Figure S2. Images of commercial test strips for detection of hCG in the concentration of 0 ng/mL and 500 ng/mL, respectively.

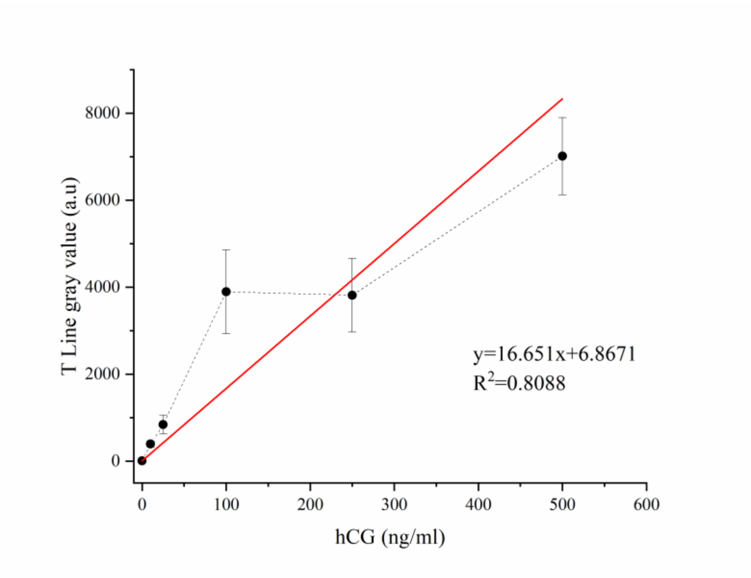

Figure S3. A calibration curve drawn of T line gray values in the range of 0 to 500 ng/mL.

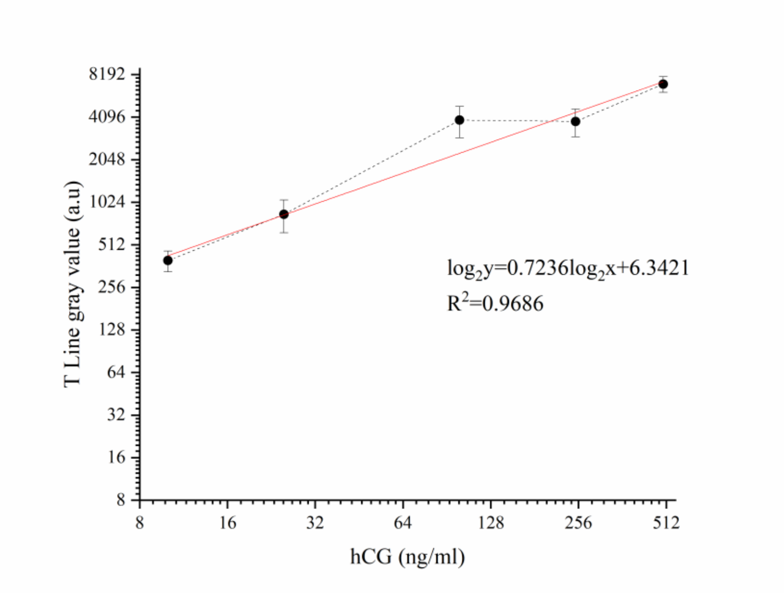

Figure S4. A calibration curve X&Y axis were calculated by double logarithmic coordinates.
